# A Pareto-Optimal Refinement Method for Protein Design Scaffolds

**DOI:** 10.1371/journal.pone.0059004

**Published:** 2013-04-02

**Authors:** Lucas Gregorio Nivón, Rocco Moretti, David Baker

**Affiliations:** 1 Department of Biochemistry, University of Washington, Seattle, Washington, United States of America; 2 Howard Hughes Medical Institute (HHMI), University of Washington, Seattle, Washington, United States of America; University of Michigan, United States of America

## Abstract

Computational design of protein function involves a search for amino acids with the lowest energy subject to a set of constraints specifying function. In many cases a set of natural protein backbone structures, or “scaffolds”, are searched to find regions where functional sites (an enzyme active site, ligand binding pocket, protein – protein interaction region, etc.) can be placed, and the identities of the surrounding amino acids are optimized to satisfy functional constraints. Input native protein structures almost invariably have regions that score very poorly with the design force field, and any design based on these unmodified structures may result in mutations away from the native sequence solely as a result of the energetic strain. Because the input structure is already a stable protein, it is desirable to keep the total number of mutations to a minimum and to avoid mutations resulting from poorly-scoring input structures. Here we describe a protocol using cycles of minimization with combined backbone/sidechain restraints that is Pareto-optimal with respect to RMSD to the native structure and energetic strain reduction. The protocol should be broadly useful in the preparation of scaffold libraries for functional site design.

## Introduction

There has been recent progress in the computational design of functional proteins and in the prediction of biomolecular interactions across a wide range of problems: ligand-protein [1] and protein-protein docking [2]; protein engineering for enzyme activity [3], [4], [5]; protein engineering for protein-protein interaction specificity control [6]; and the design of entirely novel protein folds with artificial sequences [7], [8]. In most cases of computational design for novel function one begins with the structure of an existing protein backbone “scaffold” and proceeds to remodel a local pocket or interface, or to carry out more extensive backbone or loop re-configurations, in order to meet a set of functional constraints. In the prediction of interactions one also relies upon an accurately-scored target structure for ranking of inter-molecular interactions.

Most crystal structures will have regions of high energy as evaluated in Rosetta or other design programs, which will lead to sequence changes in design if they are not addressed. However most minimization protocols will lead to too much deviation from the original wild-type crystal structure. The question is how to properly balance energy minimization with reduction of structural deviation from the starting structure. The concept of optimizing a structure to the energy function in use has a long precedent – for example the equilibration of structures in molecular dynamics prior to a production run [9]. Various methods, including torsion-space sidechain minimization, torsion-space backbone minimization, and re-sampling of sidechain rotamers (“packing”), have been applied to prepare protein structures for computational design. Recent work has shown that a protocol consisting of cycles of packing and all-atom minimization, called “fast-relax”(henceforth “relax”), is able to achieve very low energies for a given pdb quite rapidly [10]. However, the relax protocol often shifts the backbone of a protein by more than 1 Å RMSD, leading to considerable sequence changes in design. A method that reduces energetic strain while minimizing structural deviation – and the accompanying design sequence changes – would be of considerable use.

Here we carry out a systematic examination of a number of structure refinement methods, evaluating them for optimality (minimization of Rosetta energy and RMSD from the starting structure simultaneously) and for their influence on subsequent sequence re-design. We found that a combination of harmonic backbone and sidechain coordinate restraints minimizes all-atom RMSD and Rosetta energy together, and reduces the number of sequence changes in subsequent design. Because the restrained relax protocol examined here results in fewer sequence alterations in design, it also minimizes the amount of human intervention required in the overall design process.

## Results

### All-atom harmonic-restrained relax lies along a Pareto-optimal frontier between RMSD and Rosetta energy

Our goal in evaluating structure minimization protocols was first to minimize RMSD to the native (*primum non nocere*, or “first do no harm”) while also minimizing the Rosetta energy. One possibility in preparing structures for Rosetta is to re-refine the structure using an energy function incorporating standard Rosetta score terms as well as the correspondence to electron density [11]. In the general case, however, the structure factors are not available (deposition of structure factors in the PDB has only been mandatory since 2008), so it is convenient to use the input pdb coordinates as a proxy. We tested sidechain coordinate restraints with different functional forms and parameters against sidechain-sidechain pairwise distance restraints (assigning a pairwise distance restraint to any pair of sidechain atoms within a given distance). We also compared this to an electron density restrained relax protocol [11] for the 36 protein subset of the test set where density was available.

**Figure 1:**
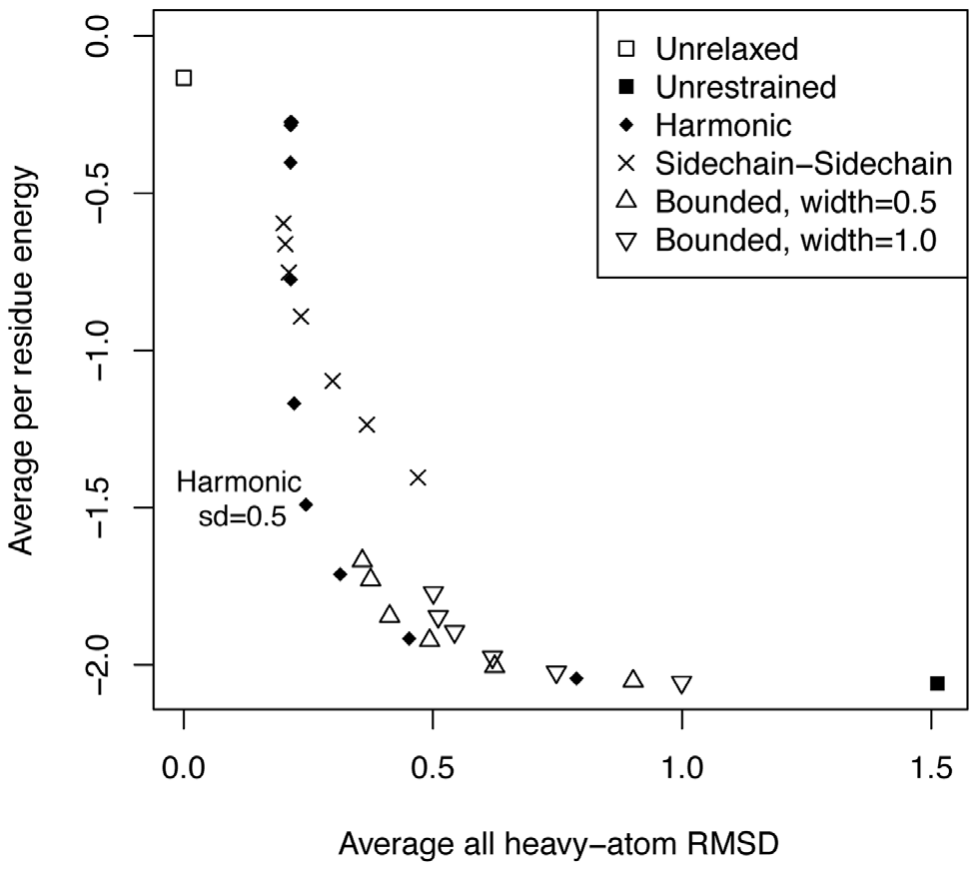
Restraint-protocol evaluation. Average all-atom RMSD to the original structure versus per residue energies across the 51 protein test set for relax runs using different restraint parameters. The relax protocol conducts cycles of sidechain repacking and all-atom minimization while ramping the repulsive energy up and down – the protocol without additional restraints is the “unrestrained” set. The harmonic set adds all-atom restraints using a harmonic potential with a width specified by the sd parameter. The bounded sets employ restraints with a zero value within a set “width” parameter and a linear value specified by an sd term further from the origin. The sidechain-sidechain restraints place harmonic restraints between pairs of sidechain atoms at a specified cutoff distance, which can be varied to produce more or less restrictive restraints.

To determine how the various parameters affect the energy/RMSD trade-off, a set of relax runs were performed on a set of 51 test structures (Figure****1). In general, the looser the restraints, the lower in energy the structure becomes, albeit with a corresponding larger structural deviation, with harmonic being Pareto optimal. Bounded all-atom restraints (with a flat center region and linear side-lobes), while approximating harmonic ones at higher restraint values, as expected have greater structural variance for tight restraints. While sidechain-sidechain distance restraints approximate the performance of all-atom harmonic restraints when tight (high cutoff value), they give higher energies for smaller restraint cutoff values. Of the harmonic restraints, sd = 0.5 gives the best energy (−1.49 REU/residue), while staying about as close to the input structure as tighter harmonic restraints (0.03 RMSD unit difference between sd = 0.5 and sd = 0.000001 sets); any larger sd value leads to larger deviations from the input structure. All-atom restraints are necessary for atomistic recapitulation, as backbone-only harmonic restraints result in all-atom RMSDs of greater than 1 Å, regardless of strength. The residual all-atom RMSD of ca. 0.21 Å in even the tightest sidechain restraint protocols is due to sidechain conformational flipping of histidine, asparagine and glutamine residues. The positioning of the sidechains of H, N and Q residues in x-ray crystal structures is somewhat ambiguous, due to near-equal electron density of the sidechain heavy atoms. While crystallographers place the conformations using best estimates, these are sometimes incorrect [12]. In these relax runs, Rosetta was instructed to consider the alternate conformation, and may not have converged to the exact heavy atom positioning in the input structure.

The relax runs were also examined for consistency between different proteins, and within multiple runs for the same protein. Most of the 51 proteins individually showed similar curves to the aggregate one in Figure****1. In all cases, the sd = 0.5 was near the knee of the curve, giving the lowest energy for minimal RMSD change. Within each protein, replicates of the harmonic sd = 0.5 procedure gave convergent results, with much more convergence for the restrained sets than the unrestrained set. The observed range of RMSDs to the input structure among ten replicates are mostly less than 0.002 Å, and all less than 0.01Å, and for the per residue energies are mostly less than 0.01 REU, and are all less than 0.11Å. In contrast, in the unrestrained relax protocol the RMSD ranges were generally over 100× greater, and the per residue energy ranges 25× greater.

### All-atom coordinate-restrained relax leads to improved sequence recovery in design

The influence of the relax protocols on natural sequence recovery in design calculations was investigated (Table****1). The relax protocol was run on each protein in the set and 50 independent sequence design runs were carried out on each structure (using the “enzyme design” procedure in Rosetta [13]). Relax with harmonic backbone and sidechain coordinate restraints with sd = 0.5 (protocol 5 in Table****1) slightly improves sequence recovery while keeping all-atom RMSD closer to the input structure than any other protocol. The addition of more rotamer sampling does not improve RMSD but does slow the calculation roughly six-fold (protocol 6). All other protocols result in larger RMSD and sequence recovery, even the sidechain-sidechain pairwise restraint (protocol 4). For the remainder of the manuscript we will refer to the all-atom harmonic-restrained relax protocol with sd = 0.5 as “restrained relax”, and we focus all subsequent analysis on this protocol.

**Table 1:** RMSD after restrained relax with various restraint protocols and sequence recovery after Rosetta design.

Pre-treatment Protocol	Protocol Number	Seq. rec. in design	All-atom RMSD (Å)	Energy per residue (after relax)
No relax	1	0.397	0	−0.36
relax no restraint	2	0.534	0.956	−2.13
relax + backbone restraint (BB)	3	0.471	1.002	−1.98
relax + BB + Sidechain-sidechain distance restraint (BB+scsc)	4	0.432	0.338	−1.60
relax + BB + Sidechain coordinate restraint (BB + sccoord)	5	0.431	0.176	−1.54
Relax + ex rotamers (6x slower)	6	0.436	0.176	−1.55

Sequence recovery is the mean over 50 separate runs. Calculations were performed with Rosetta r48056, no hydrogen-bond flags in relax, and using score12_w_corrections for relax. All protocols use the relax algorithm with various added restraints, indicated by the protocol number and description.

We also prepared a 36-protein subset of the full test set with the relax protocol using restraints to electron density instead of restraints derived from the input atomic positions (Table****2). Over this set the relax with electron density produces larger all-atom RMSD and higher per-residue energies than the all-atom restrained relax protocol. In subsequent ligand design the sequence recovery on the electron density relaxed set is only very slightly improved over the input set, while recovery for the coordinate restrained protocol is significantly higher. This suggests that the coordinate restrained protocol has some residual bias toward the input sequence.

**Table 2:** RMSD after restrained relax, and sequence recovery after Rosetta design over a 36-protein subset using electron density data.

Pre-treatment Protocol	Protocol Number	Seq. rec. in design	All-atom RMSD (Å)	Energy per residue (after relax)
No relax	1	0.389	0	−0.61
relax no restraint	2	0.511	1.512	−2.13
relax + BB + electron density	7	0.391	0.441	−1.15
relax + BB + sccoord	5	0.418	0.176	−1.62

The same tests as in Table 1 were run on the 36-protein subset for which electron density was available, but the less-optimal relax restraint protocols were not tested for this smaller set. Density data were taken from the http://eds.bmc.uu.se/eds/server.

### Characteristics of restrained-relax structures: Better rotamer probability and atom-atom repulsion energies; fewer high-energy residues

To identify the energetic changes brought about by the restrained relax protocol, we compare the various Rosetta energy terms between the input structures and the restrained relaxed structures (Table****S1 in File S1). A handful of terms contribute the vast majority of the change, with the largest contributions being from poor rotamer energies in the input structures (fa_dun) and repulsion between atoms that are slightly too close as judged by Lennard-Jones repulsion (fa_rep).

Most residues in an unmodified structure have slightly negative Rosetta energy, while a handful have positive or very positive energy, which is largely eliminated by the restrained relax protocol (Figure****S1 in File S1). We examined the structure of the putrescine receptor from *E. coli* (1A99.pdb) to understand energetic changes effected by the protocol at a residue-by-residue level. The high-energy residues about 5 REU are all moved to lower energy by the protocol; residues in the 1–5 REU range are largely moved to lower energy; residues with negative energy remain mostly unchanged (Figure****S1 in File S1). We ranked individual-residue improvements, showing the top 10 in Table****S2 in File S1. They mostly have a large decrease in either fa_dun or fa_rep after the protocol, except T161, which improves in both.

Restrained relax improves sequence recovery in design partly by minimizing the energy of poorly-scoring input residues. We looked for cases where the native residue is replaced by a new residue when the un-relaxed input structure is used, but is correctly placed when using the restrained relax structure. One such case is the rat intestinal fatty-acid-binding protein (2ifb.pdb). The design protocol on the native places a glutamate at the position 14, in place of native tyrosine (Figure****S2 and Table****S3 in File S1). The relax protocol lowers the score at this position, primarily the fa_dun. After restrained relax the design protocol leaves the native tyrosine – design will now not place a glutamate because that would increase energy above that of the native tyrosine – thus avoiding an unnecessary sequence change.

### The restrained relax protocol also increases sequence recovery in a monomeric protein design benchmark

To test the broader applicability of this protocol beyond design for ligand binding we ran the restrained relax protocol on a 41 protein sequence recovery set used to test protocols for the sequence redesign of an entire protein [14]. The protocol increases sequence recovery in the monomer benchmark from 0.371 to 0.394. Sequence recovery jumps to 0.47 if no restraints are applied, echoing the above finding that looser restraints lead to higher sequence recovery and possible over-fitting to the input structures. The mean total energies drop from 341.31 to −113.94 after the restrained relax, although it is worth noting in this case that a number of the unmodified input structures have very high Lennard-Jones (fa_rep) repulsive energies – for example, on structure has an energy over 15,000 from such a clash. RMSD calculations show CA RMSD of 0.064 Å and all-heavy-atom RMSD of 0.156 Å to the natives, averaged over all structures in the benchmark set.

## Discussion

All-atom harmonic-restrained relax strikes an optimum between energy minimization and fit to the input crystallographic data (Figure****1). Un-relaxed structures have significant deficiencies that negatively impact subsequent design. In particular, a small subset of residues is scored to have very high energy in Rosetta. The relax protocol alone, without structure-based restraints, results in relatively large structural changes (over 1 Å), which manifest in subsequent design as over-fitting to the input system and very high apparent increases in sequence recovery. By relaxing with relatively tight harmonic restraints on all heavy atoms, we can relieve the majority of the energy deficiencies (Figure****S1 in File S1) in a structure without significantly perturbing its coordinates. Over a benchmark of small, monomeric proteins, this protocol results in very small structural changes along with large energy improvements and an increase in sequence recovery in subsequent sequence design.

The more conformational freedom allowed during a relax optimization, the higher the sequence recovery in subsequent sequence design (Table****1). Sequence recovery in the enzyme design test set increases from 0.39 in the un-optimized crystal structure set to 0.53 in the set optimized with unrestrained relax. In the monomer test set sequence recovery increases from 0.37 to 0.47 after unrestrained relax. However, relax with electron density data – for the subset where the density is available – leads to only a very small increase in sequence recovery. The various restrained relax protocols may potentially over-fit the input structures to their native sequence slightly, preventing sequence changes during design. This over-optimization is undesirable, because the user typically needs to redesign the starting structure to have some new activity, and an over-optimized structure may resist sequence changes. The harmonic-restrained relax protocol minimizes this effect while removing high-energy trouble spots.

The all-atom harmonic-restrained relax protocol is Pareto-optimal when compared with a variety of different restraint methods within the relax protocol (Figure****1) [10]. Overall energies are high with a very tight harmonic restraint, and these energies rapidly drop as the restraint is broadened, with relatively little increase in RMSD until an inflection point near sd = 0.5. For any value of sd>0.5, decreases in energy are only achieved along with an increase in RMSD. The range near sd = 0.5 therefore is an optimal along the Pareto front, the position in parameter space where no further improvements can be won without a worsening in RMSD. This simple protocol should find routine use in preparing crystal structures for use in Rosetta.

## Methods

All Rosetta runs were performed with svn revision 48056, which corresponds to the Rosetta 3.4 public release.

The 51 test protein-ligand complexes were from the enzyme design benchmark set of high-resolution ligand binding structures (manuscript in preparation, Nivón and Bjelic). Briefly, these structures were prepared for Rosetta by removal of waters and non-canonical amino acids and preparation of Rosetta parameter files for the ligands. Structure selection is described fully in Nivón and Bjelic.

The 41 protein monomer sequence recovery set has been described elsewhere [14]. All runs of design on this set use a fixed Monte Carlo seed to keep all parameters repeatable.

The fast relax protocol in Rosetta is described elsewhere [10]. Briefly, it consists of five cycles with rotamer repacking and minimization with progressively higher repulsive contributions within each cycle. Restraints are added to the structure prior to the start of the protocol, and contribute to the energy function over which the protein is repacked and minimized. By default, restraints are turned off with increasing repulsive contributions, but setting “-relax:ramp_constraints false” on the command line can preserve the restraints throughout the entire protocol. This setting is used in cases where restraints are experimentally derived, such as for electron density. The running time for the all-atom restrained relax protocol is roughly exponential in the number of residues, with a 200 residue protein taking approximately 10****min on one core of a mixed Intel-Xeon-L5335-2GHz/AMD-Opteron-2.2GHz cluster and roughly doubling in time for each additional 100 residues.

Harmonic coordinate restraints take the form f(x)  =  (d/sd)?2, where d is the distance of the atom from the desired coordinate, and sd is a parameter related to the strength of the restraint. Bounded coordinate restraints take a zero value within WIDTH of the desired coordinate, followed by a small harmonic segment to transition (from WIDTH to WIDTH +0.5*sd) and a linear value of slope set at 1/sd for the rest. Sidechain-sidechain restraint runs have harmonic distance restraints of sd = 2.0 for all sidechain atom pairs within the specified cutoff distance, and backbone heavy atom harmonic coordinate restraints of sd = 0.5.

Parameter scans were performed with the flags:

“-no_optH false -flip_HNQ -use_input_sc -correct -no_his_his_pairE -linmem_ig 10 -nblist_autoupdate true”

Harmonic runs added flags “-constrain_relax_to_start_coords -relax:ramp_constraints false -relax:coord_constrain_sidechains -relax:coord_cst_stdev <SD>”, where SD ranged from 0.000001 to 5.0

Bounded runs added flags “-constrain_relax_to_start_coords -relax:ramp_constraints false -relax:coord_constrain_sidechains -relax:coord_cst_stdev <SD> -relax:coord_cst_width <WIDTH>”, where SD ranged from 0.1 to 5.0, and WIDTH ranged from 0 to 1.0.

Sidechain-sidechain runs added flags “-constrain_relax_to_start_coords

-relax:ramp_constraints false -relax:sc_cst_maxdist <DIST>” where DIST ranged from 3 to 8.

Unrestrained runs had no additional flags.

RMSD values were computed in pymol with the command “align relaxed and not hydro, reference and not hydro, cycles = 0”. The median per-residue energy and RMSD of 10 replicates of the relax procedure were taken as the value for that protein, and the mean value across the 51 proteins in the input set were computed for each of the different parameter runs.

Design was performed with the Rosetta enzyme design program, using the default parameters from the enzdes scientific test in rosetta (rosetta/rosetta_tests/scientific/biweekly/tests/enzdes_benchmark). Briefly, a design shell of 6 Å, with a 12 Å shell of repackable (rotamer changes allowed, but no sequence changes) residues was used. One cycle of design with a “soft” Lennard Jones repulsive term was followed by one with the standard repulsive term. Extra rotamers were included based on the Dunbrack rotamer distribution, and recent updates to the Rosetta score function were also included [15] (hydrogen bond corrections from Leaver-Fay and O'Meara, unpublished results).

### Design flags

–l./inputs/pdb.list

–enzdes::detect_design_interface

–enzdes::cut1 6.0

–enzdes::cut2 8.0

–enzdes::cut3 10.0

–enzdes::cut4 12.0

–enzdes::cst_design

–enzdes::design_min_cycles 2

–enzdes::cst_min

–enzdes::chi_min

–ex1

–ex2

–ex1aro

–ex2aro

–extrachi_cutoff 1

–soft_rep_design

–flip_HNQ

–correct

–no_his_his_pairE

–score::hbond_params correct_params

–lj_hbond_hdis 1.75

–lj_hbond_OH_donor_dis 2.6

–dun08 false

–nstruct 1

–enzdes::no_unconstrained_repack

–linmem_ig 10

–nblist_autoupdate true

–enzdes::lig_packer_weight 1.8

–docking::ligand::old_estat true

–extra_res_fa inputs/2b3b.params inputs/2ifb.params inputs/1sw1.params inputs/2FQX.params inputs/2p0d.params inputs/2DRI.params inputs/1fby.params inputs/1ZHX.params inputs/2RDE.params inputs/1db1.params inputs/2h6b.params inputs/1z17.params inputs/2FME.params inputs/1y3n.params inputs/1urg.params inputs/1FZQ.params inputs/1y52.params inputs/1POT.params inputs/1XT8.params inputs/2FR3.params inputs/2UYI.params inputs/1USK.params inputs/1n4h.params inputs/2qo4.params inputs/2GM1.params inputs/2rct.params inputs/2HZQ.params inputs/1hsl.params inputs/1A99.params inputs/1uw1.params inputs/1l8b.params inputs/3B50.params inputs/1H6H.params inputs/2Q2Y.params inputs/1hmr.params inputs/1OPB.params inputs/1x7r.params inputs/2Q89.params inputs/1nl5.params inputs/1TYR.params inputs/2e2r.params inputs/1LKE.params inputs/2PFY.params inputs/1wdn.params inputs/1nq7.params inputs/1y2u.params inputs/2ioy.params inputs/1J6Z.params inputs/1RBP.params inputs/1XZX.params inputs/2f5t.params

–chemical:exclude_patches LowerDNA UpperDNA Cterm_amidation SpecialRotamer

VirtualBB ShoveBB VirtualDNAPhosphate VirtualNTerm CTermConnect sc_orbitals

pro_hydroxylated_case1 pro_hydroxylated_case2 ser_phosphorylated

thr_phosphorylated tyr_phosphorylated tyr_sulfated lys_dimethylated

lys_monomethylated lys_trimethylated lys_acetylated glu_carboxylated

cys_acetylated tyr_diiodinated N_acetylated C_methylamidated

MethylatedProteinCterm

## Supporting Information

File S1 **Supporting figures and tables. Table S1.** Energy comparison between input and restrained-relax structures. Average over all energy terms for the input set (INPUT) and the set relaxed with sidechain coordinate restraints at sd = 0.5 (COORD). Values are sorted by the difference in each score term. Energy terms fa_dun (sidechain rotamer probability-based energy) and fa_rep (Lennard-Jones repulsive term) are the largest contributors to lower energy after the relax. **Table S2.** The top-10 most-improved residues after the relax protocol for 1A99. Score term changes are shown for fa_dun and fa_rep score terms only. INPUT is for the original wild-type crystal structure. COORD is for the coordinate restrained relax structures. Delta is the difference between INPUT and COORD for the specified score term and residue. **Table S3.** Residue 14 energies for 2ifb.pdb before/after restrained relax and before/after design. Note that after the restrained relax the total energy for residue 14 (−1.17) is much lower, even slightly lower than the energy after enzyme design on an unrelaxed input structure (−1.14). **Figure S1.** Per-residue score comparison between an input and restrained-relax structure. Histogram of individual residue Rosetta scores for the putrescine receptor (1A99.pdb) unmodified (blue) and after the coordinate-restrained relax protocol (red), with overlap in purple. **Figure S2.** Example of improved design after restrained relax. 2ifb position 14 example, native in green, design on the unmodified native in cyan, and design on the restrained-relax native in purple. The wild-type identity at position 14 is mutated from tyrosine to glutamate in the run on an unmodified structure (cyan), but remains the original tyrosine in the run on the restrained-relax structure (purple).
(DOCX)
