# Supplementary material for: A Pareto-Optimal Refinement Method for Protein Design Scaffolds
Source: PLoS One. 2013 Apr 2;8(4):e59004. doi: 10.1371/journal.pone.0059004 (PMC3614904; doi:10.1371/journal.pone.0059004)
Supplement: File S1 — Supporting figures and tables. Table S1. Energy comparison between input and restrained-relax structures. Average over all energy terms for the input set (INPUT) and the set relaxed with sidechain coordinate restraints at sd = 0.5 (COORD). Values are sorted by the difference in each score term. Energy terms fa_dun (sidechain rotamer probability-based energy) and fa_rep (Lennard-Jones repulsive term) are the largest contributors to lower energy after the relax. Table S2. The top-10 most-improved residues after the relax protocol for 1A99. Score term changes are shown for fa_dun and fa_rep score terms only. INPUT is for the original wild-type crystal structure. COORD is for the coordinate restrained relax structures. Delta is the difference between INPUT and COORD for the specified score term and residue. Table S3. Residue 14 energies for 2ifb.pdb before/after restrained relax and before/after design. Note that after the restrained relax the total energy for residue 14 (−1.17) is much lower, even slightly lower than the energy after enzyme design on an unrelaxed input structure (−1.14). Figure S1. Per-residue score comparison between an input and restrained-relax structure. Histogram of individual residue Rosetta scores for the putrescine receptor (1A99.pdb) unmodified (blue) and after the coordinate-restrained relax protocol (red), with overlap in purple. Figure S2. Example of improved design after restrained relax. 2ifb position 14 example, native in green, design on the unmodified native in cyan, and design on the restrained-relax native in purple. The wild-type identity at position 14 is mutated from tyrosine to glutamate in the run on an unmodified structure (cyan), but remains the original tyrosine in the run on the restrained-relax structure (purple). (DOCX) [file pone.0059004.s001.docx]

**A Pareto-Optimal Refinement Method for Protein Design Scaffolds**

**Lucas Gregorio Nivón, Rocco Moretti, David Baker**

**Supporting Information**

**Table S1**

| **Energy term name** | **INPUTS (mean)** | **COORD (mean)** | **Delta** |
| --- | --- | --- | --- |
| fa_dun | 404.19 | 245.01 | -159.18 |
| fa_rep | 207.59 | 117.73 | -89.86 |
| fa_cust_pair_dist | 27.55 | 1.21 | -26.34 |
| omega | 35.57 | 26.20 | -9.37 |
| ch_bond_bb_bb | -19.50 | -27.90 | -8.40 |
| hbond_bb_sc | -77.28 | -84.50 | -7.22 |
| hbond_sc | -25.29 | -31.98 | -6.69 |
| hbond_lr_bb | -60.53 | -67.02 | -6.48 |
| fa_sol | 531.28 | 527.77 | -3.50 |
| rama | 7.05 | 6.02 | -1.04 |
| pro_close | -14.67 | -15.51 | -0.85 |
| p_aa_pp | -17.86 | -18.66 | -0.80 |
| fa_pair | 0.88 | 0.39 | -0.49 |
| dslf_ss_dst | -0.96 | -1.16 | -0.20 |
| hack_elec | 2.48 | 2.32 | -0.16 |
| dslf_cs_ang | 0.18 | 0.05 | -0.13 |
| hbond_sr_bb | -21.29 | -21.41 | -0.12 |
| fa_atr | -1051.68 | -1051.78 | -0.10 |
| dslf_ss_dih | 0.13 | 0.08 | -0.06 |
| ref | -19.69 | -19.69 | 0.00 |
| dslf_ca_dih | 0.01 | 0.01 | 0.00 |
| coordinate_constraint | 0.00 | 60.16 | 60.16 |
| total | -91.84 | -352.66 | -260.82 |
| total_minus_coord | -91.84 | -412.81 | -320.98 |

**Table S2.**

| **label** | **total INPUT** | **total COORD** | **fa_dun INPUT** | **fa_dun COORD** | **fa_rep INPUT** | **fa_rep COORD** | **Delta Total** | **Delta fa_dun** | **Delta fa_rep** |
| --- | --- | --- | --- | --- | --- | --- | --- | --- | --- |
| V284 | 19.51 | 0.76 | 3.61 | 1.72 | 18.26 | 1.39 | -18.75 | -1.89 | -16.87 |
| Y286 | 17.38 | -0.29 | 1.34 | 1.60 | 19.94 | 2.49 | -17.67 | 0.26 | -17.45 |
| K188 | 13.95 | 0.34 | 15.61 | 1.54 | 0.43 | 0.26 | -13.61 | -14.08 | -0.17 |
| T161 | 12.90 | 0.01 | 5.12 | 0.69 | 9.78 | 0.39 | -12.89 | -4.43 | -9.39 |
| E157 | 7.92 | -1.20 | 1.48 | 1.59 | 10.16 | 0.85 | -9.13 | 0.11 | -9.31 |
| V212 | 6.91 | -1.20 | 1.92 | 0.67 | 7.81 | 0.69 | -8.11 | -1.25 | -7.12 |
| L144 | 4.54 | -2.35 | 0.17 | 0.34 | 7.87 | 0.76 | -6.89 | 0.17 | -7.11 |
| V129 | 6.82 | 1.32 | 6.74 | 1.39 | 1.97 | 1.32 | -5.50 | -5.35 | -0.64 |
| E156 | 5.16 | -0.20 | 8.50 | 2.28 | 0.27 | 0.43 | -5.36 | -6.22 | 0.16 |
| S131 | 4.75 | -0.07 | 4.65 | 0.35 | 2.47 | 0.98 | -4.82 | -4.29 | -1.49 |

**Table S3.**

| **Protocol** | **fa_dun** | **Total** |
| --- | --- | --- |
| No relax (Y) | 3.0 | 0.41 |
| Restrained relax (Y) | 1.34 | -1.17 |
| No relax after enzdes (E) | 1.31 | -1.14 |
| Restrained relax after enzdes (Y) | 0.48 | -1.62 |


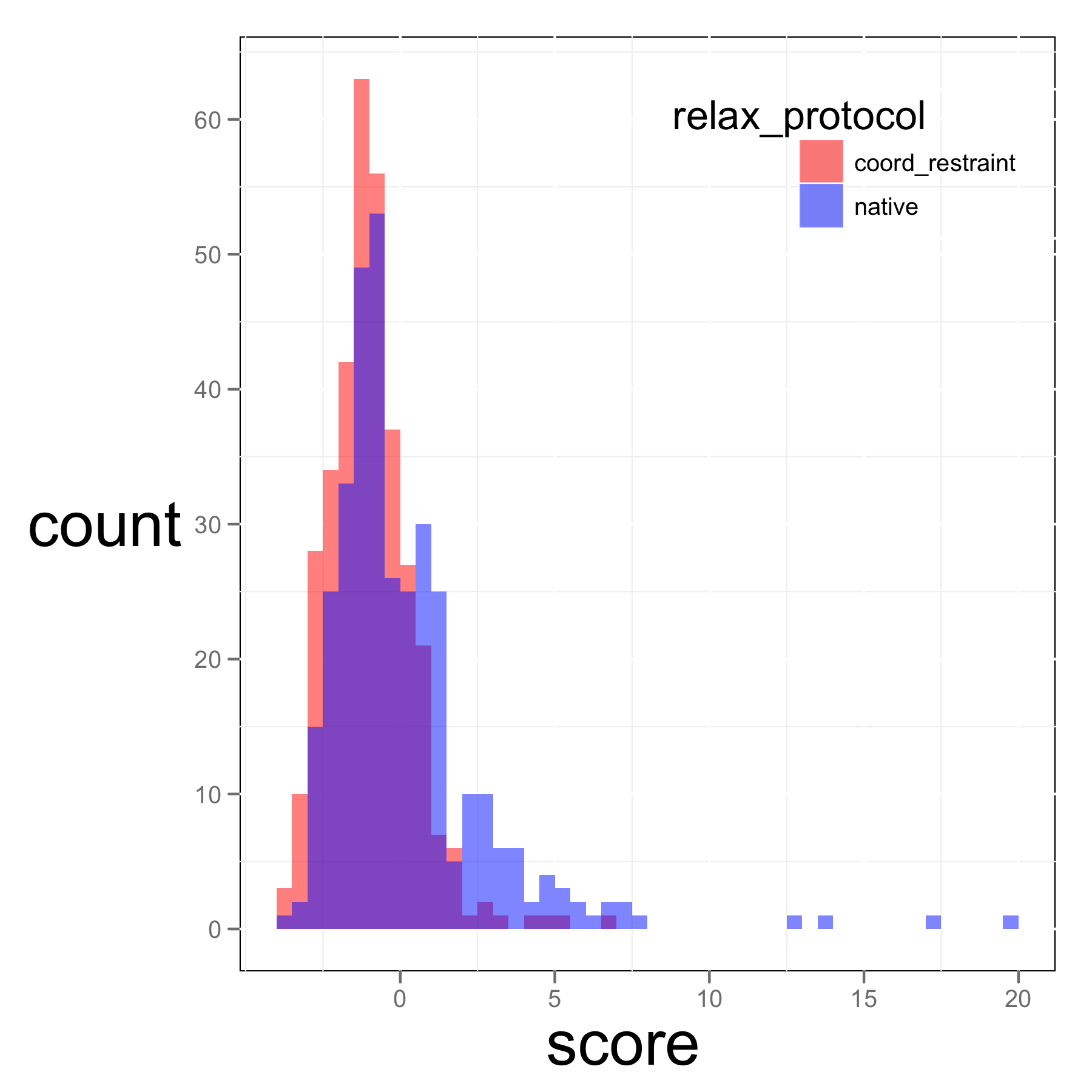


**Figure S1.**


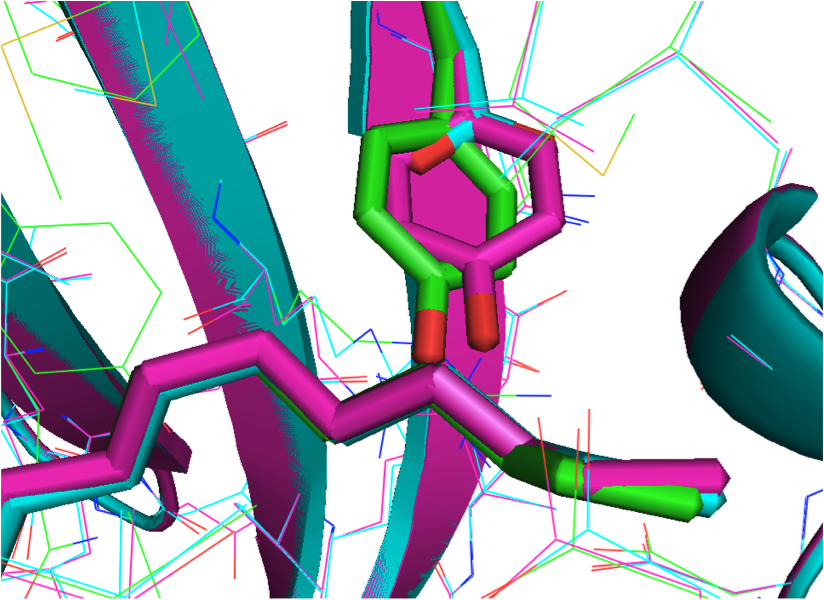


**Figure S2.**

**Release info**

Special issue requirements: We have integration test, demo and documentation:

rosetta_source/doc/apps/public/prepare_pdb_for_rosetta_with_relax.dox

rosetta/rosetta_tests/integration/tests/relax_w_allatom_cst

/work/nivon/rosetta/rosetta_demos/public/prepare_pdb_for_rosetta_with_relax
